# The role of branched-chain amino acids and their downstream metabolites in mediating insulin resistance

**DOI:** 10.3389/jpps.2024.13040

**Published:** 2024-06-28

**Authors:** Abdualrahman Mohammed Abdualkader, Qutuba G. Karwi, Gary D. Lopaschuk, Rami Al Batran

**Affiliations:** ^1^ Faculty of Pharmacy, Université de Montréal, Montréal, QC, Canada; ^2^ Montreal Diabetes Research Center, Montréal, QC, Canada; ^3^ Cardiometabolic Health, Diabetes and Obesity Research Network, Montréal, QC, Canada; ^4^ Division of BioMedical Sciences, Faculty of Medicine, Memorial University of Newfoundland, St. John’s, NL, Canada; ^5^ Mazankowski Alberta Heart Institute, University of Alberta, Edmonton, AB, Canada

**Keywords:** BCAAs, BCKAs, obesity, insulin resistance, type 2 diabetes

## Abstract

Elevated levels of circulating branched-chain amino acids (BCAAs) and their associated metabolites have been strongly linked to insulin resistance and type 2 diabetes. Despite extensive research, the precise mechanisms linking increased BCAA levels with these conditions remain elusive. In this review, we highlight the key organs involved in maintaining BCAA homeostasis and discuss how obesity and insulin resistance disrupt the intricate interplay among these organs, thus affecting BCAA balance. Additionally, we outline recent research shedding light on the impact of tissue-specific or systemic modulation of BCAA metabolism on circulating BCAA levels, their metabolites, and insulin sensitivity, while also identifying specific knowledge gaps and areas requiring further investigation. Finally, we summarize the effects of BCAA supplementation or restriction on obesity and insulin sensitivity.

## Introduction

Branched chain amino acids (BCAAs) are a group of three indispensable amino acids: leucine, isoleucine and valine. Together, they account for approximately 35% of the essential amino acids present in the human body. While the primary source of BCAAs is dietary intake [1], certain bacteria within the gut microbiome are capable of synthesizing them as well [2, 3]. However, the degree to which the gut microbiome produces BCAAs varies among individuals and is influenced by factors such as diet, gut microbiome composition, and overall health. Apart from serving as fundamental components in protein synthesis, BCAAs, especially leucine, play a critical role in stimulating protein synthesis through the activation of the mechanistic target of rapamycin (mTOR) signalling pathway [4]. Elevated plasma concentrations of BCAAs have been observed in both obese individuals and animal models of obesity [5–9]. Although plasma levels of other amino acid may also rise in obesity, the elevation in BCAAs are of particular interest because they appear to have unique effects in obesity-induced insulin resistance, and they are considered a major contributor to the pathology of type 2 diabetes (T2D) and coronary artery disease [10]. Numerous studies since the 1960s have consistently linked elevated plasma BCAAs with insulin resistance [7]. Furthermore, a landmark metabolomics profiling study even suggests that elevation in circulating BCAA levels can predict insulin resistance and T2D as much as 20 years prior to clinical presentation and a decade before any other known marker or test [11]. Interestingly, gastric bypass surgery in obese patients, which effectively lowers elevated BCAA levels, correlates with improved glucose homeostasis and enhanced insulin sensitivity [12]. Despite extensive research efforts, the underlying mechanisms by which elevated BCAA levels contribute to the development of insulin resistance and T2D remain unclear. In this Review, we highlight the major organs responsible for BCAA homeostasis. We then delve into how obesity and insulin resistance affect the communication between these organs, thereby influencing the maintenance of BCAA homeostasis. We also outline recent studies that sheds light on how modulating BCAA metabolism, either in a tissue-specific manner or at a whole-body level, impact circulating BCAA levels and their downstream metabolites, and the consequent effects on obesity and insulin resistance. We end by summarizing the effects of BCAA supplementation or restriction on obesity and insulin sensitivity.

## Overview of BCAA catabolism

**FIGURE 1:**
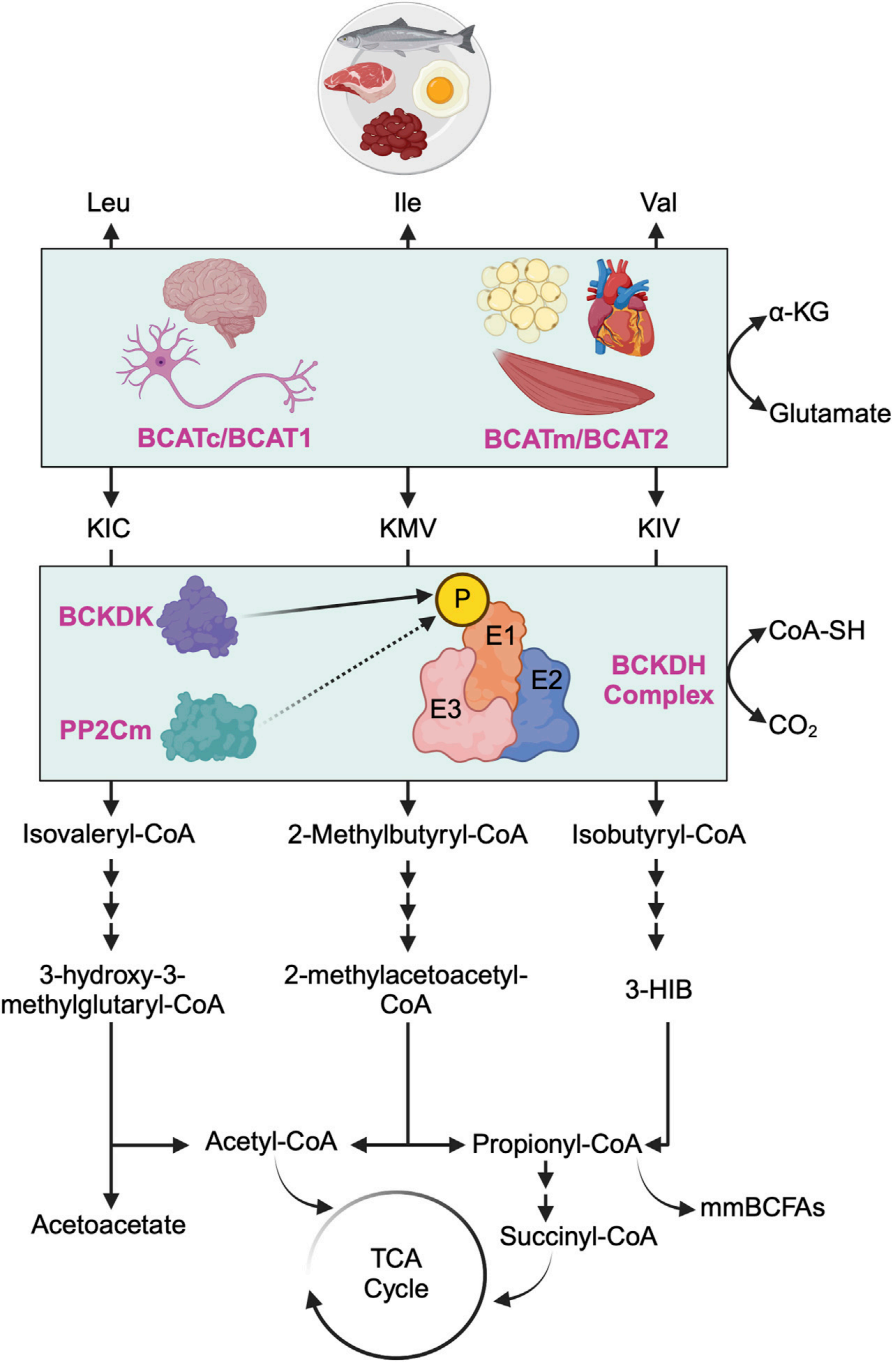
Overview of branched-chain amino acid catabolism pathway. The initial and shared step in the catabolism of all three branched-chain amino acids (BCAAs) - leucine (Leu), isoleucine (Ile), and valine (Val) - involves the reversible transamination of BCAAs to produce branched-chain alpha-ketoacids (BCKAs). Specifically, Leu yields α-ketoisocaproate (KIC), Ile yields α-keto-ß-methylvalerate (KMV), and Val yields α-ketovalerate (KIV). This transamination process is catalyzed by two distinct isoforms of branched-chain amino acid aminotransferase (BCAT): the cytosolic isoform (BCATc/BCAT1, encoded by the *Bcat1* gene), predominantly found in the central nervous system and peripheral nerves, and the mitochondrial isoform (BCATm/BCAT2, encoded by the *Bcat2* gene), primarily located in the mitochondria of most nonneuronal tissues. Subsequently, all three BCKAs (KIC, KMV, and KIV) undergo irreversible oxidative decarboxylation, facilitated by the branched-chain alpha-ketoacid dehydrogenase (BCKDH) complex, which serves as the rate-limiting enzyme in BCAA oxidation. The BCKDH complex comprises of three components: E1 (encoded by *Bckdha* and *Bckdhb* genes), E2 (encoded by *Dbt* gene), and E3 (encoded by *Dld* gene). The activity of the BCKDH complex is tightly regulated by BCKDH kinase (BCKDK), which phosphorylates E1 of the BCKDH complex and inhibits its activity (i.e., inhibiting BCAA oxidation), whereas protein phosphatase 2Cm (PP2Cm) dephosphorylates E1 of the BCKDH complex and activates its activity (i.e., activating BCAA oxidation). Post-decarboxylation, each BCKA follows a distinct metabolic pathway, generating acyl-CoA derivatives (isovaleryl-CoA from KIC, 2-methylbutyryl-CoA from KMV, and isobutyryl-CoA from KIV) and various downstream metabolites. These metabolites include critical metabolic intermediates for the TCA cycle, such as acetyl-CoA or succinyl-CoA, as well as acetoacetate, metabolic end products of Leu catabolism, 3-hydroxyisobutyrate (3-HIB), a downstream metabolite of Val that stimulates fatty acid uptake, and monomethyl branched-chain fatty acids (mmBCFAs), adipocyte-specific metabolites derived from mitochondrial BCAA catabolism, namely, propionyl-CoA.

Plasma BCAA levels at the whole-body level are regulated by a delicate balance between input factors, such as dietary protein intake and proteolysis, and output factors, encompassing protein synthesis and oxidation. Insulin plays a pivotal role in maintaining this balance. Under normal and healthy conditions, insulin facilitates the cellular uptake of BCAAs while suppressing proteolysis, thus regulating plasma BCAA concentrations. However, in pathological states like insulin resistance, this regulatory mechanism may be disrupted. For instance, research on obese women has indicated that moderate obesity correlates with heightened proteolysis and impaired anti-proteolytic effects of insulin [13]. Another study suggested that the increased proteolysis observed in obesity and insulin resistance may be attributed to the compromised anti-proteolytic function of insulin [14]. The intricate regulatory relationship between insulin and BCAA metabolism has been extensively explored in previous literature reviews [15–17]. To facilitate a comprehensive understanding for the reader, we begin by providing essential information on BCAA catabolism and oxidation before delving into the role of BCAAs in mediating insulin resistance (Figure 1). The initial step in the BCAA catabolic pathway involves the reversible transamination of BCAAs catalyzed by branched-chain amino acid aminotransferase (BCAT). Notably, there exist two distinct isoforms of BCAT, namely, BCAT1 encoded by the cytosolic gene (*Bcat1*) and BCAT2 encoded by the mitochondrial gene (*Bcat2*). BCAT1 is the less common of the two isoforms and is primarily expressed in the cytoplasm, with a notable presence in the central and peripheral nervous systems [18, 19], while BCAT2 is the more ubiquitous isoform found in the mitochondria of most nonneuronal tissues, such as the heart, kidney, skeletal muscle and adipose tissue, excluding the liver [20, 21]. BCAT transfers the amino group from BCAAs to α-ketoglutarate, producing glutamate and the corresponding branched-chain α-keto acids (BCKAs): α-ketoisocaproate (KIC) from leucine, α-keto-β-methylvalerate (KMV) from isoleucine, and α-ketoisovalerate (KIV) from valine. This transamination reaction generates ammonia as a byproduct, particularly in the muscles. To remove excess ammonia, the muscle activates the alanine cycle (also known as the Cahill cycle), converting pyruvate to alanine by attaching the amino group from glutamate to pyruvate. Additionally, muscles convert glutamate and ammonia to glutamine as another means of ammonia detoxification. Both alanine and glutamine, as non-toxic carriers of ammonia, are transported to the liver, where the ammonia can be further processed and excreted [22, 23].

Following BCAAs transamination, the irreversible oxidative decarboxylation of BCKAs is catalyzed by the branched-chain α-ketoacid dehydrogenase (BCKDH) complex, serving as the rate-limiting step in BCAA oxidation. The BCKDH complex comprises three components: E1 (encoded by *Bckdha* and *Bckdhb* genes, functioning as a thiamine-dependent decarboxylase), E2 (encoded by *Dbt* gene, functioning as dihydrolipoyl transacylase), and E3 (encoded by *Dld* gene, functioning as dihydrolipoamide dehydrogenase) [17]. The activity of the BCKDH complex is tightly regulated by BCKDH kinase (BCKDK), which phosphorylates and inhibits the BCKDH complex, and protein phosphatase 2Cm (PP2Cm), responsible for dephosphorylating and activating the BCKDH complex [24]. After decarboxylation, each BCKA follows a distinct metabolic route, ultimately leading to the formation of either acetyl-CoA or succinyl-CoA for energy production in the tricarboxylic acid (TCA) cycle or other metabolic intermediates such as acetoacetate, 3-hydroxyisobutyrate (3-HIB) or monomethyl branched-chain fatty acids (mmBCFAs).

## Major organs responsible for BCAA homeostasis

BCAA metabolism is an intricate process that relies on inter-organ communication to maintain BCAA homeostasis. Among the key contributors to the circulating pool of BCAAs, skeletal muscle emerges as a predominant site. Skeletal muscle plays a pivotal role in BCAA transamination, primarily owing to the substantial abundance of BCAT2 within the muscle and its considerable muscle mass [19]. Importantly, skeletal muscle not only serves as a hub for BCAA transamination but also stands out as a major site for BCAA oxidation (accounting for 59% of whole-body BCAA oxidation) and protein synthesis (contributing to 24% of the total protein synthesis from BCAAs) [16]. In contrast, the liver does not engage in BCAA transamination or BCKA re-amination due to the lack of BCAT2 in hepatocytes [15]. Instead, owing to the liver’s high BCKDH activity, BCKAs derived from BCAA transamination in extrahepatic tissues are transported to the liver, where they can serve as substrates for BCAA oxidation [15, 25]. While BCKDH complex activity is notably high in the liver and comparatively low in adipose tissue [19, 26], recent tracing studies in mice have uncovered brown adipose tissue as an additional significant site for BCAA oxidation, constituting 19% of whole-body BCAA oxidation, followed by the liver at 8% [27]. This observation has been further supported by another study that used positron emission tomography-computed tomography scans with a leucine-analogue tracer in mice and humans. The study concluded that, upon cold exposure, brown adipose tissue, but not white adipose tissue, significantly contributes to systemic BCAA clearance by enhancing BCAA uptake in this tissue compartment to generate heat through thermogenesis [28].

Numerous studies have demonstrated that inter-organ communication essential for maintaining BCAA homeostasis is disturbed in obesity and insulin resistance. For example, studies showed that in two different rodent models of obesity and insulin resistance (*ob/ob* mice and Zucker rats), the BCKDH activity is decreased in the liver [29–31]. Additionally, other studies have consistently revealed reductions in BCKDH complex expression or activity in white adipose tissue across various models of obesity and insulin resistance [32, 33]. Remarkably, transplanting white adipose tissue from wild-type mice into BCAT2 or PP2CM deficient mice has been found to lower circulating BCAA levels [32, 34], highlighting the pivotal role of adipose tissue in regulating BCAA levels systemically. In line with these findings, Neinast and colleagues uncovered that in *db/db* mice, a model of severe insulin resistance, BCAA oxidation is impaired in adipose tissues and liver and redirected towards skeletal muscle [27]. The same group also demonstrated that excess BCAA oxidation in skeletal muscle leads to the secretion of 3-HIB, a downstream metabolite of valine, which, in turn, stimulates muscle fatty acid uptake and lipid accumulation, thereby exacerbating insulin resistance [35]. Another group hypothesized that in obesity and insulin resistance, the accumulation of C3 and C5 acylcarnitines in muscle, which are by-products of BCAA catabolism and markers of incomplete fat oxidation, may contribute to insulin resistance [36, 37]. A recent hypothesis posits a direct association between BCKAs and insulin resistance, where exposure of muscle cells to high concentrations of BCKAs results in the inhibition of insulin-induced AKT phosphorylation (also known as protein kinase B) and glucose uptake [38], indicating a direct role of BCKAs in impairing insulin signalling. Finally, the classical mechanism linking elevated BCAA levels with insulin resistance involves chronic hyperactivation of mTORC1 and its downstream effector, ribosomal protein S6 kinase 1 (S6K1), also known as p70-S6K. This hyperactivation phosphorylates and inhibits insulin receptor substrate 1 (IRS-1), thus blunting insulin signalling and contributing to insulin resistance [39–41].

Indeed, the role of the gut microbiome in maintaining BCAA homeostasis was historically overlooked due to the complexity of the microbiome, technological limitations, and a traditional focus on host genetics and diet. However, recent advances in this domain have highlighted the microbiome’s critical role in BCAA synthesis, regulation, and interaction with host metabolism. It is now evident that the gut microbiome contributes to the overall pool of BCAAs, potentially influencing the development of insulin resistance [42]. For example, a landmark study identified *Bacteroides vulgatus* and *Prevotella copri* as two key species of gut microbiome bacteria responsible for elevated BCAA biosynthesis and associated with insulin resistance in humans [3]. This study also demonstrated that *Prevotella copri* can induce insulin resistance, exacerbate glucose intolerance, and increase circulating levels of BCAAs in mice. Furthermore, a recent study demonstrated that feeding mice a variety of protein sources mirroring the composition of the Western diet exacerbates insulin resistance. This effect is attributed to an increase in gut microbial branched-chain fatty acids (BCFA) [43], a class of short-chain fatty acids produced in the gut through the proteolytic fermentation of BCAAs.

Nevertheless, in the subsequent sections, we will discuss and summarize the effects of modifying BCAA catabolism, either selectively in a tissue-specific manner (muscles, liver, adipose tissue, and heart) or systemically, on circulating BCAA levels and insulin sensitivity (Table 1)

**TABLE 1:** The effects of modulating BCAA catabolism in various tissue compartments or systemically on insulin sensitivity in lean and obese animals.

Study design	Outcome	References
Muscle		
Muscle-specific *Bckdk* knockout mice fed a chow diet	• Increased muscle BCAA oxidation • Decreased plasma BCAA and BCKA levels only during fasting state • No change in glucose tolerance and insulin sensitivity	[44]
Muscle-specific *Bckdk* knockout mice fed an HFD or WD	• Decreased plasma BCAA levels • No change in glucose tolerance and insulin sensitivity	[44]
Muscle-specific *Dbt* knockout mice fed a chow diet	• Decreased muscle BCAA oxidation • No change in plasma BCAA levels during both fasting and refeeding states • No change in glucose tolerance and insulin sensitivity	[44]
Muscle-specific *Dbt* knockout mice fed an HFD	• No change in plasma BCAA levels during both fasting and refeeding states • No change in glucose tolerance and insulin sensitivity	[44]
Liver		
Overexpressing *Ppm1k* in the liver of Zucker fatty rats	• Increased liver BCAA oxidation • Decreased plasma BCAA levels • Improved glucose tolerance and insulin sensitivity	[45]
Liver-specific *Bckdk* knockout mice fed a chow or HFD	• Increased liver BCAA oxidation • No change in plasma BCAA levels during both fasting and refeeding states • No change in insulin sensitivity	[44]
Liver-specific *Dbt* knockout mice fed a chow or HFD	• Decreased liver BCAA oxidation • No change in plasma BCAA levels during both fasting and refeeding states • No change in insulin sensitivity	[44]
Muscle- and liver-specific *Bckdk* knockout mice fed an HFD	• Increased muscle and liver BCAA oxidation • No change in plasma BCAA levels during fasting state • No change in insulin sensitivity	[44]
Liver-specific *Bcat2* transgenic mice fed an HFD	• No change in plasma BCAA levels • Impaired glucose tolerance	[46]
Adipose Tissue		
BAT-specific *Bckdha* knockout mice fed an HFD	• Impaired BCAA clearance • Susceptible to HFD-induced obesity and insulin resistance • Impaired BAT BCAA and glucose oxidation	[28]
WAT-specific *Bcat2* knockout mice fed an HFD	• Increased plasma BCAA levels • Resistance to HFD-induced obesity and insulin resistance • BCKAs supplementation restore obesity and insulin resistance	[47]
Heart		
Heart-specific *Bcat2* knockout mice	• Decreased heart BCAA oxidation • Increased cardiac BCAAs and decreased BCKAs • Increased cardiac insulin sensitivity	[48]
*Ppm1k* knockout mice	• Decreased systemic BCAA oxidation • Increased plasma BCAA and BCKA levels • Sensitized the heart to ischemia-reperfusion injury	[49]
*Ppm1k* knockout mice	• Decreased systemic BCAA oxidation • Increased plasma BCAA and BCKA levels • Promoted heart failure	[50]
Systemic		
*Bcat2* knockout mice fed an HFD	• Increased plasma BCAAs and decreased BCKAs • Improved glucose tolerance and insulin sensitivity • Increased energy expenditure	[51]
*Bckdk* knockout mice fed a chow diet	• Increased systemic BCAA oxidation • Decreased plasma BCAA and BCKA levels • No change in glucose tolerance	[27]
*Ppm1k* knockout mice fed a chow diet or HFD	• Decreased systemic BCAA oxidation • Increased plasma BCAA and BCKA levels • Improved glucose tolerance and insulin sensitivity	[52, 53]
Zucker fatty rats treated with LY3351337	• Increased plasma BCAA and glycine levels • Improved glucose tolerance and insulin sensitivity	[54]
Obese and insulin-resistant animals treated with Telmisartan	• Decreased plasma BCAA levels • Improved glucose tolerance and insulin sensitivity	[47]
Obese and insulin-resistant animals treated with BT2	• Increased systemic BCAA oxidation • Decreased plasma BCAA and BCKA levels • I mproved glucose tolerance and insulin sensitivity	[27, 44, 45, 55, 56]

HFD, high-fat diet; WD, western diet; BAT, brown adipose tissue; WAT, white adipose tissue; BCAA, branched-chain amino acid; BCKA, branched-chain α-keto acid; *Bckdk*, branched-chain keto acid dehydrogenase kinase; *Dbt*, dihydrolipoamide branched-chain transacylase E2; *Ppm1k*, protein phosphatase, Mg^2+^/Mn^2+^. Dependent 1K; *Bcat2*, branched-chain amino acid transaminase 2; *Bckdha*, branched-chain keto acid dehydrogenase E1 subunit alpha; LY3351337, BCAT1 and BCAT2 inhibitor; Telmisartan, BCAT2 inhibitor; BT2, BCKDK, inhibitor.

## Modulating BCAA catabolism to treat insulin resistance

*Muscle:* Skeletal muscle plays a crucial role in maintaining BCAA homeostasis, serving as the primary site for whole-body BCAA oxidation. In Zucker-fatty rats, BCKDH activity is elevated in skeletal muscle but reduced in the liver compared to Zucker-lean rats [29]. Similarly, Neinast and colleagues demonstrated that in *db/db* mice, but not in mice fed a high-fat diet for 14 weeks, BCAA oxidation is increased in skeletal muscle and decreased in the liver and adipose tissue [16, 27]. Furthermore, several studies have noted diminished BCAA oxidation in adipose tissues during obesity and insulin resistance [30, 32]. These collective observations from multiple research groups have led to the hypothesis that excess BCAA oxidation in skeletal muscle may contribute to insulin resistance. This may occur via two potential mechanisms: 1) through the overproduction of 3-HIB in muscle or 2) via the accumulation of acylcarnitines derived from muscle BCAA breakdown. In both scenarios, this would impair fatty acid oxidation and promote lipotoxicity [29, 37, 57]. To test this hypothesis, Blair et al [44] generated muscle-specific knockout mice lacking either the *Bckdk* gene, responsible for phosphorylating E1 of the BCKDH complex and inhibiting its activity, or the *Dbt* gene, crucial for BCAA oxidation as it encodes the E2 component of the BCKDH complex. Interestingly, their investigation revealed that augmenting muscle BCAA oxidation lowered plasma BCAA and BCKA levels only during the fasting state in muscle-specific *Bckdk* knockout mice fed a chow diet compared to their control littermates, human α-skeletal actin (HSA)-Cre mice. Conversely, diminishing muscle BCAA oxidation did not significantly alter plasma BCAA levels during both fasting and refeeding states in muscle-specific *Dbt* knockout mice fed a chow diet compared to HSA-Cre mice. However, the administration of a single bolus of BCAA resulted in impaired BCAA and BCKA clearance in muscle-specific *Dbt* knockout mice when compared to their controls. These findings indicate that manipulating muscle BCAA oxidation under healthy conditions impacts circulating BCAA levels predominantly during fasting.

To investigate whether modulating muscle BCAA oxidation impacts glucose homeostasis, muscle-specific *Bckdk* knockout mice were subjected to chronic feeding regimens of either a Western diet or a high-fat diet spanning from 4 up to 12 weeks, followed by assessment of insulin sensitivity and glucose handling using hyperinsulinemic-euglycemic clamp and glucose tolerance tests (GTT), respectively. Surprisingly, despite observing a reduction in plasma BCAAs and an increase in the 3-HIB/valine ratio during the fasted state in muscle-specific *Bckdk* knockout mice fed the obesogenic diet (Western diet or high-fat diet), this alteration did not manifest in changes in insulin sensitivity or glucose handling. Specifically, there were no discernible differences in euglycemic clamp and GTT outcomes between muscle-specific *Bckdk* knockout mice and their controls. Similarly, inhibiting muscle BCAA oxidation in muscle-specific *Dbt* knockout mice fed a high-fat diet did not affect insulin sensitivity during a euglycemic clamp or alter glucose handling during a GTT. Additionally, there were no significant changes observed in plasma BCAA levels or the 3-HIB/valine ratio in both fasted and refed states. These collective findings suggest that augmenting or diminishing muscle BCAA oxidation has no impact on whole-body insulin sensitivity in mice subjected to various obesogenic diets. While this study did not directly measure it, further exploration into the effects of modulating muscle BCAA oxidation on muscle insulin sensitivity itself would be intriguing. Moreover, investigating whether muscle acylcarnitine species, particularly C3 and C5, as well as BCKA levels play a role in improving or exacerbating muscle insulin sensitivity could provide valuable insights.

*Liver:* While the liver lacks the BCAT enzyme necessary for the conversion of BCAAs into BCKAs and *vice versa*, it remains a pivotal site for BCAA oxidation and protein synthesis, contributing up to 27% of whole-body BCAA incorporation into proteins [27]. As previously noted, multiple studies have demonstrated markedly elevated expression of liver BCKDK, which phosphorylates and inhibits BCKDH complex activity, in various models of obese and insulin-resistant rodents [29–31]. In an effort to understand whether the reduction in liver BCAA oxidation contributes to the development of insulin resistance, White and colleagues utilized adenovirus-mediated delivery of *Ppm1k*, the gene encoding PP2Cm (which dephosphorylates and activates the BCKDH complex), to specifically overexpress PP2Cm in the liver of Zucker fatty rats [45]. Their findings demonstrated that liver PP2Cm overexpression enhanced liver BCKDH activity, reduced circulating BCAAs, alleviated hepatic steatosis, and improved glucose tolerance and insulin sensitivity. Interestingly, hepatic overexpression of PP2Cm increased the phosphorylation of ATP-citrate lyase (ACLY), a critical enzyme involved in lipid synthesis. This activation of ACLY subsequently stimulated *de novo* lipogenesis, thereby integrating BCAA metabolism with lipid metabolism.

On the contrary, manipulating liver BCAA oxidation levels through targeting either the *Bckdk* or *Dbt* gene in the liver, using an adeno-associated viral (AAV) vector carrying the Cre recombinase gene under the control of the thyroxine-binding globulin (TBG) promoter (AAV8-TBG-Cre) in *Bckdk* or *Dbt* floxed mice, did not influence circulating BCAA levels in either fasted or refed states, regardless of whether the mice were subjected to a chow or high-fat diet for 4–5 weeks [44]. Furthermore, neither enhancing nor suppressing liver BCAA oxidation affected whole-body insulin sensitivity in mice fed a normal chow or high-fat diet. Of note, augmenting both muscle and liver BCAA oxidation in mice, achieved by treating muscle-specific *Bckdk* knockout mice with AAV8-TBG-Cre to generate double knockout mice, also failed to impact whole-body insulin sensitivity in mice subjected to a high-fat diet for 6 weeks. This was observed despite a notable reduction in fasting plasma BCAAs and an increase in the 3-HIB/valine ratio in BCKDK double knockout mice compared to their control counterparts. Although the reasons for the discrepancy between the results regarding the manipulation of liver BCAA oxidation and insulin sensitivity in the mouse and rat studies remain unclear, it has been suggested that species differences may account for the contrasting outcomes [57]. Of note, a recent study revealed that mice lacking PP2Cm globally are protected against high-fat-diet-induced insulin resistance. Interestingly, this investigation also demonstrated that BCKAs selectively inhibits the mitochondrial pyruvate carrier (MPC) in hepatocytes, thus suppressing gluconeogenesis from pyruvate [52]. Nevertheless, further research is warranted to delineate the role of liver BCAA catabolism in insulin resistance.

*Adipose tissue:* Adipose tissue, traditionally viewed as a passive site for energy storage, is now recognized as a dynamic regulator impacting various aspects of whole-body metabolism, including BCAA catabolism. In conditions like obesity and insulin resistance, there is a notable suppression in the expression of nearly all enzymes responsible for BCAA catabolism, particularly within white adipose tissue [30, 32, 55, 58]. This decrease in BCAA catabolism is considered a significant contributor to the systemic elevation of BCAA levels during obesity and insulin resistance [33, 59]. Cross-tissue flux studies comparing lean and healthy individuals to insulin-sensitive or insulin-resistant obese subjects revealed negligible uptake of BCAAs from human abdominal subcutaneous white adipose tissue [33]. However, BCAA catabolic enzyme levels were markedly reduced in omental fat, a specific type of visceral fat, but not in subcutaneous white adipose tissue of obese individuals with metabolic syndrome compared to weight-matched healthy obese subjects. This finding suggests that alterations in BCAA catabolism in visceral white adipose tissue significantly contribute to the BCAA metabolic phenotype in individuals with insulin resistance. Furthermore, adipose tissue not only utilizes BCAAs to support adipocyte differentiation and lipogenesis [60], but it also has the capacity to release adipocyte-specific metabolites stemming from mitochondrial BCAA catabolism, such as mmBCFAs. These metabolites play a role in fueling *de novo* lipogenesis, with their levels being notably decreased in obese animals and increased during prolonged fasting [61]. Consequently, it is tempting to speculate that the reduced levels of plasma mmBCFAs observed in obesity may be attributed to decreased BCAA catabolism within this specific tissue compartment.

Adipose tissue, particularly brown adipose tissue, plays a significant role in utilizing BCAAs for thermogenesis during cold exposure in both mice and humans. This process contributes to systemic BCAA clearance by enhancing BCAA uptake via SLC25A44, a mitochondrial BCAA transporter [28]. Notably, BCAA clearance following oral administration of BCAAs is compromised in mice with targeted deletion of *Bckdha* in brown adipose tissue, the gene responsible for encoding the E1 component of the BCKDH complex and critical for BCAA oxidation. Moreover, brown adipose tissue-specific *Bckdha* knockout mice showed increased susceptibility to high-fat diet-induced obesity and insulin resistance, coupled with impaired glucose oxidation within brown adipose tissue. These findings underscore the critical role of intact BCAA oxidation in brown adipose tissue for systemic BCAA clearance and the amelioration of obesity and insulin resistance. Conversely, a recent study indicates that white adipose tissue-specific *Bcat2* knockout mice display resistance to high-fat diet-induced obesity and insulin resistance, attributed to enhanced browning and thermogenesis in white adipose tissue [47]. Intriguingly, the study also revealed that BCKAs inhibits white adipose tissue browning through the acetylation of the PR domain-containing protein 16 (PRDM16). Furthermore, supplementation of BCKAs in white adipose tissue-specific *Bcat2* knockout mice reverses these favorable effects, leading to the reinstatement of obesity and insulin resistance. These findings suggest that mitigating BCAA transamination into BCKAs in white adipose tissue, consequently affecting BCAA oxidation in this tissue compartment, is beneficial in attenuating obesity and insulin resistance.

One pivotal question emerges from the findings of these two studies: Why is the suppression of BCAA oxidation in brown adipose tissue detrimental rather than protective against insulin resistance, whereas its suppression in white adipose tissue appears to confer a protective effect? One potential explanation for this phenomenon is that inhibiting BCAA oxidation in brown adipose tissue not only raises BCAA levels but also elevates the level of BCKAs, thereby triggering insulin resistance. Conversely, targeting BCAT2 in white adipose tissue leads to increased BCAA levels while concurrently reducing BCKAs, thus mitigating insulin resistance. In support of this hypothesis, mice with whole-body *Bcat2* deletion exhibit elevated plasma BCAAs and decreased BCKAs, yet remained protected from high-fat diet-induced obesity and insulin resistance [51]. Remarkably, this protection persists even in the presence of mTORC1 hyperactivation, as evidenced by the phosphorylation of mTOR downstream targets such as eukaryotic translation initiation factor 4E (eIF4E)-binding protein 1 (4E-BP1) and S6K1 in the gastrocnemius of *Bcat2* knockout fasted mice. Collectively, these findings suggest that it may not be the mere elevation of circulating BCAAs *per se* that drives insulin resistance, but rather the accumulation of BCKAs that plays a crucial role in mediating insulin resistance. Given that skeletal muscle predominantly facilitates the conversion of BCAAs into BCKAs, it would be interesting to explore whether decreasing BCKAs, particularly in muscle tissue, by targeting BCAT2 yields outcomes akin to reducing BCKAs in white adipose tissue. Future studies are imperative to answer this question.

*Heart:* Decreased cardiac BCAA oxidation has been linked to the development of cardiac insulin resistance and impaired cardiac insulin signalling pathways [62]. Direct measurement of cardiac BCAA oxidation rates in isolated working mouse hearts demonstrated that these rates are decreased in a mouse model of high-fat diet-induced obesity [63]. This is associated with a decreased activity of Akt and glycogen synthase kinase-3β (GSK-3β) and cardiac insulin-stimulated glucose oxidation rates in obese mice [63]. Similarly, cardiac BCAA oxidation rates are also decreased in the failing heart, which is associated with impaired insulin signalling and insulin-stimulated glucose oxidation rates [64]. A whole-body PP2Cm deletion, a maneuver which decreases the activity of BCKDH complex and BCAA oxidation, is associated with decreased glucose oxidation by inhibiting pyruvate dehydrogenase (PDH) activity and increased vulnerability to myocardial ischemia/reperfusion injury [49].

Since impaired BCAA oxidation leads to the accumulation of BCAAs and BCKAs, it is difficult to ascertain whether BCAAs or BCKAs contribute to cardiac insulin resistance. Selective increasing cardiac BCKA levels abrogates insulin-stimulated cardiac glucose oxidation rates via inhibiting insulin signalling pathway *ex vivo* [48]. While BCKAs could be re-aminated to their correspondent BCAAs [65], it is unclear how fast this process is. In fact, we recently demonstrated that an acute increase in BCKA does not lead to a significant change in cardiac BCAA levels. Moreover, we recently developed a mouse model where we deleted BCAT2 in the heart to selectively increase cardiac BCAAs and decrease cardiac BCKAs [48]. The accumulation of cardiac BCAA levels in the *Bcat2* knockout hearts did not impact cardiac insulin sensitivity [48]. However, BCAT2 deletion enhances cardiac insulin signalling and insulin-stimulated glucose oxidation rates [48]. These findings demonstrate that it is BCKAs, not BCAAs, that influence cardiac insulin signalling. In further support of this, we recently showed that reducing cardiac BCKA levels by cardiac-specific deletion of *Bcat2* mitigates cardiac insulin resistance and enhances insulin-stimulated glucose oxidation rates in the failing heart [66]. This enhancement in cardiac glucose oxidation is mediated, at least in part, via enhancing mitochondrial Akt activity [66]. How BCKAs enhance insulin signalling in the heart remains an interesting scope for future investigations.

*Systemic:* At the whole-body level, the use of LY3351337 to inhibit both BCAT1 and BCAT2 in Zucker fatty rats results in increased circulating levels of BCAA and glycine [54], with the latter showing an inverse correlation with impaired glucose handling and T2D [67, 68]. This intervention significantly improves glucose tolerance and insulin sensitivity. Similarly, inhibition of BCAT2 with Telmisartan reduces circulating BCKA levels and body weight, leading to notable enhancements in glucose tolerance and insulin sensitivity in mice on a high-fat diet [47]. Furthermore, a plethora of studies utilizing various animal models of obesity and insulin resistance consistently demonstrates that treatment with the BCKDK inhibitor 3,6-dichlorobenzo[b]thiophene-2-carboxylic acid, commonly referred to as BT2, enhances BCAA oxidation, reduces circulating BCAA and BCKA levels, and notably improves glucose tolerance and insulin sensitivity [27, 44, 45, 55]. In a recent randomized and controlled clinical trial, T2D patients receiving sodium phenylbutyrate (4.8g per day), an FDA-approved drug for treating acute hyperammonemia that inhibits BCKDK and promotes BCAA oxidation, exhibited enhanced insulin sensitivity after just 2 weeks of treatment compared to the placebo group [69]. It is worth mentioning that enhancing systemic BCAA oxidation through global deletion of BCKDK in lean mice results in decreased circulating BCAA and BCKA levels [27]. However, this manipulation does not significantly impact glucose disposal in BCKDK knockout lean mice following oral glucose administration. Alternatively, suppressing whole-body BCAA oxidation in mice by deleting PP2Cm increases circulating BCAA and BCKA levels and enhances glucose tolerance and insulin sensitivity, regardless of the presence or absence of obesity and insulin resistance [52, 53].

One pivotal question emerges from these studies: Is it better to augment or reduce BCAA oxidation in obesity and insulin resistance? We argue that enhancing BCAA oxidation enhancing and mainly lowering BCAA and BCKA levels appears to be a more advantageous approach, given the adverse effects associated with suppressing BCAA oxidation and increasing BCAA and BCKA levels, such as ischemia-reperfusion injury and heart failure [49, 50]. Consequently, another fundamental question remains unanswered: Does systemic enhancement of BCAA oxidation alone alleviate insulin resistance, or is it the reduction in BCKAs that alleviates insulin resistance? We propose the latter based on the following evidence: **1)** While BT2 administration in animals dephosphorylates and activates the BCKDH complex in multiple organs, resulting in a systemic reduction in plasma BCAA and BCKA levels [45, 70], the magnitude of BCKA reduction appears more pronounced compared to BCAA reduction in *ob/ob* mice treated with BT2 over a period of 4–6 weeks [55]. **2)** Screening efforts aimed at discovering more potent BCKDK inhibitors led to the identification of thiophene PF-07208254 as an allosteric BCKDK inhibitor exhibiting superior potency to BT2 [56]. Both PF-07208254 and BT2 dephosphorylate BCKDH at the same site, resulting in diminished levels of BCKAs and improved glucose tolerance and insulin sensitivity. Intriguingly, structure-activity relationship studies have revealed thiazoles as BCKDK inhibitors with even greater potency than PF-07208254 and BT2. However, despite their ability to dephosphorylate BCKDH, thiazole inhibitors elevate BCKA levels and counteract the favorable effects of PF-07208254 and BT2 by increasing the proximity of BCKDK to BCKDH-E2. **3)** In individuals with maple syrup urine disease, the oxidation of BCAAs is hindered due to a deficiency in BCKDH enzyme. It’s noteworthy that despite elevated plasma BCAA levels, these individuals do not typically experience insulin resistance [71–74].

Together, these observations further suggest that primarily reducing systemic levels of BCKAs may enhance insulin sensitivity. However, further research in this area is needed to thoroughly investigate and confirm this hypothesis.

## The impact of dietary BCAA supplementation or restriction on insulin resistance

Numerous preclinical studies have indicated that supplementing with BCAAs worsens insulin resistance, while restricting their intake improves insulin sensitivity in various obese animal models (Table 2). Recent evidence further suggests that limiting dietary BCAAs could potentially improve health and longevity in male mice [83], whereas high BCAA consumption induces obesity and shortens lifespan in mice [84]. While many of these studies have treated all three BCAAs as having equivalent metabolic effects, emerging research indicates that each BCAA may exert unique influences on obesity and insulin sensitivity. For instance, Yu and colleagues demonstrated that restricting either isoleucine or valine, but not leucine, enhances glucose tolerance and hepatic insulin sensitivity in mice on a Western diet [81]. Intriguingly, reintroducing either isoleucine or all three BCAAs, but not leucine or valine alone, reverses these metabolic benefits. In another study, the same researchers found that lifelong isoleucine restriction increases lifespan and improves glucose homeostasis in both male and female mice [82]. Similarly, another group observed that valine supplementation in mice on a high-fat diet significantly impairs glucose tolerance and insulin sensitivity [77]. Likewise, a plethora of studies have illustrated that leucine supplementation yields various beneficial effects on glucose homeostasis across different mouse models of obesity and insulin resistance [85–91]. Notably, it is well-documented that leucine increases hypothalamic mTOR signalling while reducing food intake and body weight [92]. Collectively, these findings underscore that each of the individual BCAAs exerts distinct metabolic effects on obesity and insulin sensitivity. Furthermore, accumulating evidence suggests that the elevation of BCAA levels *per se* may not be the primary driver of insulin resistance [47, 48, 51], but rather their downstream metabolites (such as BCKAs, 3-HIB, and specific acylcarnitine species) that play a pivotal role in triggering the disease. Since each BCAA follows a distinct metabolic pathway after oxidation (Figure 1), this presents promising opportunities to selectively target either the isoleucine, valine, or both pathways to treat and prevent obesity and insulin resistance.

**TABLE 2:** Preclinical studies demonstrating the impact of dietary BCAA supplementation or restriction on insulin sensitivity.

Study design	Outcome	References
BCAA Supplementation		
Wistar rats fed an HFD supplemented with BCAAs for 13 weeks	• Increased plasma BCAA levels • Impaired glucose tolerance and insulin sensitivity • Increased muscle C3 and C5 acylcarnitine levels	[36]
Obese mice subjected to exercise with or without BCAA supplementation for 12 weeks	• BCAA supplementation increased BCAA levels in WAT • BCAA supplementation impaired insulin sensitivity • Increased adiposity after BCAA supplementation	[75]
*Ob/ob* mice fed an isocaloric low-protein diet supplemented with BCAAs for 2 weeks	• Increased plasma BCAA and BCKA levels • Impaired glucose tolerance and insulin sensitivity • Increased plasma insulin levels	[55]
Mice fed an HFHS or HFD supplemented with BCAAs for 32 weeks	• Increased plasma BCAA and BCKA levels • No change in glucose tolerance and insulin sensitivity	[76]
Mice fed an HFD supplemented with valine for 15 weeks	• Impaired glucose tolerance and insulin sensitivity	[77]
BCAA Restriction		
Zucker-fatty rats fed an isocaloric BCAA-restricted LFD for 15 weeks	• Decreased plasma BCAA levels • No change in plasma BCKA levels • Improved muscle insulin sensitivity	[29]
Mice fed a BCAA-restricted WD for 12 weeks	• Reduced body weight and adiposity • Improved glucose tolerance and insulin sensitivity • Increased energy expenditure	[78]
Mice fed a low-protein or low-BCAA diet for 3 weeks	• Decreased plasma BCAA levels • Reduced body weight and adiposity • Improved glucose and pyruvate tolerance	[79]
*Ob/ob* mice fed an isocaloric low-protein diet for 4 weeks	• Decreased plasma AA levels • Decreased plasma BCAA and BCKA levels • Improved glucose tolerance and insulin sensitivity	[55]
*Db/db* mice fed diets lacking any individual BCAAs for 1 day	• Improved insulin sensitivity	[80]
Mice fed an isoleucine- or valine-restricted WD for 12 weeks	• Reduced body weight and adiposity • Improved glucose tolerance and hepatic insulin sensitivity	[81]
Mice fed an isoleucine-restricted diet for 14 weeks	• Reduced body weight and adiposity • Improved glucose tolerance and insulin sensitivity	[82]

LFD, low-fat diet; HFD, high-fat diet; HFHS, high-fat high-sucrose; WD, western diet; BCAA, branched-chain amino acid; BCKA, branched-chain α-keto acid; AA, amino acid.

## Discussion

**FIGURE 2:**
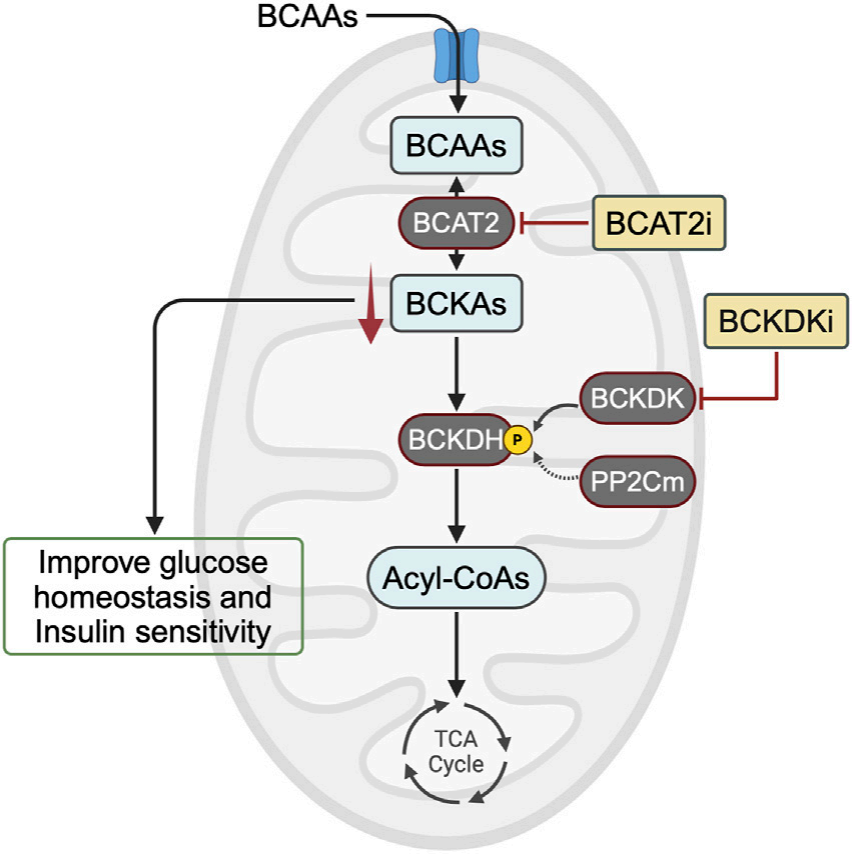
Potential targets for correcting alterations in BCAA metabolism to treat insulin resistance. In animal models of obesity and insulin resistance, mounting evidence suggests that BCKA accumulation, rather than BCAA accumulation, impairs glucose homeostasis and decreases insulin sensitivity. Therefore, one approach to lowering BCKA levels would be to inhibit BCAT2 or enhance BCAA oxidation activity using a BCKDK inhibitor. BCAA, branched-chain amino acid; BCKA, branched-chain α-keto acid; BCAT2, branched-chain amino acid aminotransferase 2; BCKDH, branched-chain α-ketoacid dehydrogenase complex; BCKDK, BCKDH kinase; PP2Cm, protein phosphatase 2Cm; BCAT2i, BCAT2 inhibitor; BCKDKi, BCKDK inhibitor.

While elevated plasma levels of BCAAs have consistently been linked to insulin resistance and T2D, recent evidence suggests that the direct implication of BCAAs themselves in insulin resistance may not be significant. Instead, emerging evidence suggests that the accumulation of their downstream metabolites, such as BCKAs, could play a crucial role in exacerbating insulin resistance. If elevated BCKA levels are indeed the main driver of insulin resistance, then lowering them can be accomplished through BCAT2 inhibition or BCKDK inhibition (Figure 2). Further research is needed to determine whether targeting these downstream metabolites of BCAAs could offer a promising avenue for treating and preventing obesity-induced insulin resistance and T2D.
